# Pre-hCG 3D and 3D power Doppler assessment of the follicle for improving pregnancy rates in intrauterine insemination cycles

**DOI:** 10.4103/0974-1208.57224

**Published:** 2009

**Authors:** Sonal Panchal, C. B. Nagori

**Affiliations:** Department of Ultrasound, Dr. Nagori's Institute for Infertility and IVF, Ahmedabad, India

**Keywords:** Cumulus, follicle assessment, pre-hCG, 3D power Doppler

## Abstract

**BACKGROUND:**

The assessment of follicular maturity at the time of human chorionic gonadotropin (hCG) is one of the key factors for the success of all assisted reproductive techniques.

**AIM:**

To assess follicles by three dimensional (3D) and 3D power Doppler (PD) before giving hCG to improve pregnancy rates in intrauterine insemination (IUI) cycles.

**DESIGN:**

Prospective randomized study.

**MATERIALS AND METHODS:**

Ultrasound for pre-hCG follicular assessment was performed over a period of 10 months for all 1000 cycles of IUI. Follicular assessment was performed using a transvaginal multifrequency volume probe. Follicles considered mature by 2D US and color Doppler were assessed by 3D and 3D PD. These values were independently evaluated for the conception and the non-conception groups.

**RESULTS:**

Conception rates were 32.3 and 27% respectively and individually when the perifollicular resistance index was < 0.50 and the peak systolic velocity was > 11 cm/s 10-12 h before hCG. Conception rates of 32% were achieved with a follicular volume between 3 and 7 cc. The conception rate was 32.3% in the cumulus group. A perifollicular vascularity index of between six and 20 gave conception rates of 35% and perifollicular flow index of 27-43 gave conception rates of 33%.

**CONCLUSIONS:**

3D ultrasound is much more accurate for volume assessment of the follicle. Presence of cumulus increases the surety of the presence of a mature ovum in the follicle. 3D and 3D PD when used with 2D US and color Doppler for pre-hCG follicular assessment would definitely improve pregnancy rates in IUI cycles.

## INTRODUCTION

The advance in ultrasound technology has changed the management of infertility. The accuracy of diagnosis and monitoring of infertility treatments such as ovulation induction has greatly increased because of the availability of sophisticated ultrasound technology and equipment.[1] Since the advent of the transvaginal ultrasound, this has been a preferred method for the assessment of the follicle and the endometrium. The assessment of follicular maturity at the time of human chorionic gonadotropin (hCG) is one of the key factors for the success of all assisted reproductive technique procedures.

### Basis of the study

The follicular size of 16-18 mm is considered as appropriate on two-dimensional (2D) ultrasound (US) for hCG administration for ovulation trigger. But, maturation of the follicle and the endometrium, ovulation and leutinization are processes of multiple biochemical, morphological and vascular changes. The vascular changes are a reflection of the biochemical changes and can be studied by color Doppler. The spectral/pulse Doppler values give an objective assessment of the follicular vascularity. Therefore, color and pulse Doppler speak about the functional maturity of the follicle and therefore the quality of the ovum. Three-dimensional (3D) US and 3D power Doppler (3D PD) assesses the global vascularity as compared to vascularity in a single plane on 2D ultrasound and so may give a better idea about follicular maturity, time of hCG and, therefore, pregnancy rates.

## MATERIALS AND METHODS

**Figures 1 and 2:**
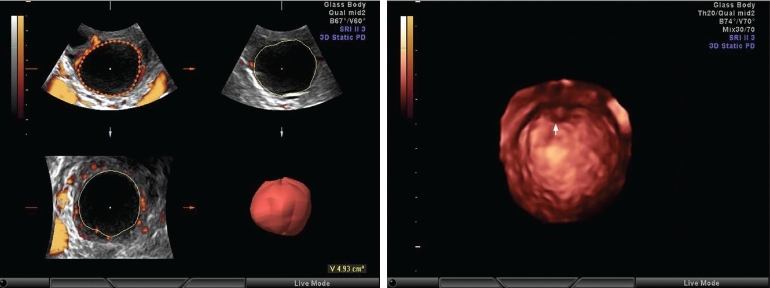
Follicular volume by the Vocal software and cumulus oophorus in the pre-ovulatory follicle

A prospective randomized study of 1000 cycles of intrauterine insemination (IUI) was performed over a period of 10 months. The various stimulation protocols for ovulation induction used were clomiphene citrate, letrozole + recombinant follicle stimulating hormone (rFSH) and only rFSH. Follicular assessment was done on Voluson E8 (Wipro GE) and all studies were performed by a single operator. A transvaginal multifrequency volume probe 6-12 MHz was used for scanning. Speckle reduction imaging (SRI) was used when required for better resolution. On 2D US, the follicular diameter was measured as a single diameter vertically on the screen when the follicle is rounded. When the follicle was polygonal due to pressure effect from adjacent follicles, the follicle was imaged in two perpendicular planes and three diameters were taken at perpendicular planes of this follicle to take an average to decide the follicular diameter. A follicle was called mature when it was round, thin walled, with no internal echoes, measuring at least 16 mm for gonadotropin stimulation and at least 18 mm for clomiphene citrate stimulation. In these follicles, cumulus- like shadows were searched for. Color Doppler assessment was performed for all these follicles to see how much of the circumference of the follicle is covered by the blood vessels. If the blood vessels were scanty on color Doppler, PD was tried to see the vascularity better. For both color and PD, the pulse repetition frequency (PRF) was set at 0.3 kHz, wall filter at the lowest, balance at 180 and gains just enough to fill up perifollicular vessels but not to spill the color outside the blood vessels. For pulse Doppler, the PRF was set to 2.2 kHz and wall filter to 60 Hz. Angle correction was performed. The vessels that overlap and obliterate the visibility of the follicular wall only were considered perifollicular vessels. After this assessment, a 3D US and 3D PD was carried out for all the patients. PRF settings are fixed at 0.3 always and the volume box is switched on. The size of the volume box was adjusted enough to include the whole follicle and at least a surrounding 5-7 mm margin. The angle of the volume was selected to be large enough to cover the whole follicle from edge to edge. The acquired volume is seen as a follicle in three perpendicular planes on the screen. Using the Vocal Software with 15° angle, the follicle is traced at its circumference at every 15° rotation and at the end the command ‘done’ is given, the region of interest (ROI) is ultimately accepted or any corrections required may be made and the computer then calculates the volume of the follicle [Figure 1].

**Figures 3 and 4:**
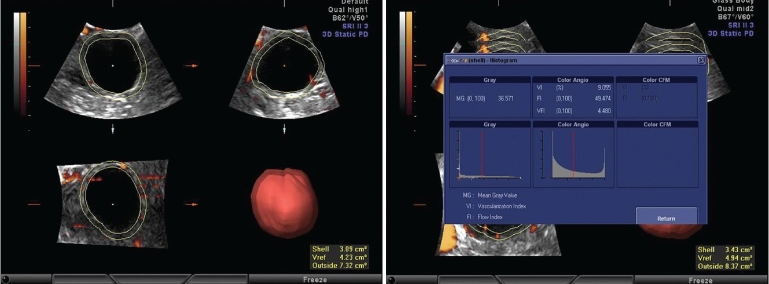
Pre hCG perifollicular 3DPD values

After the volume calculation, the follicle was seen on the rendered image. Using the surface smooth or light gradient mode for rendering with high threshold can show cumulus [Figure 2]. Then, ROI is again edited to select the option for the shell volume. An option for the outside shell with a wall thickness of 2 mm is selected, which has been found to be the most appropriate to include the perifollicular vessels. This is accepted and a volume histogram is switched on. This gives the vascularity index (VI), which calculates the abundance of flow in the selected volume, flow index (FI), which calculates the average intensity of flow in the selected volume and vascular flow index (VFI), which calculates the perfusion of the selected volume. VI indicates the abundance of the color vessels in the given volume, FI indicates the intensity of the color in the given volume and VFI is the ratio of the abundance and intensity, meaning it gives an idea about the general perfusion status of the given volume [Figures 3 and 4].

## RESULTS

**Figures 5 and 6:**
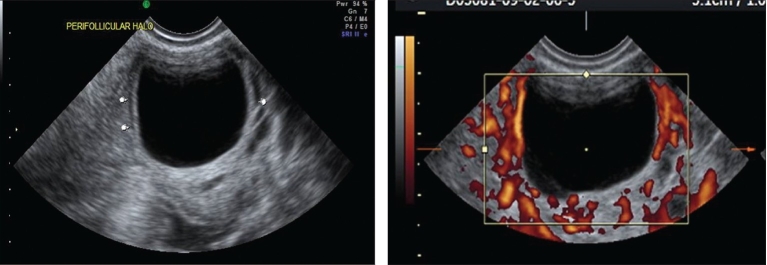
Preovulatory follicle with 2D power Doppler

**Figure 7:**
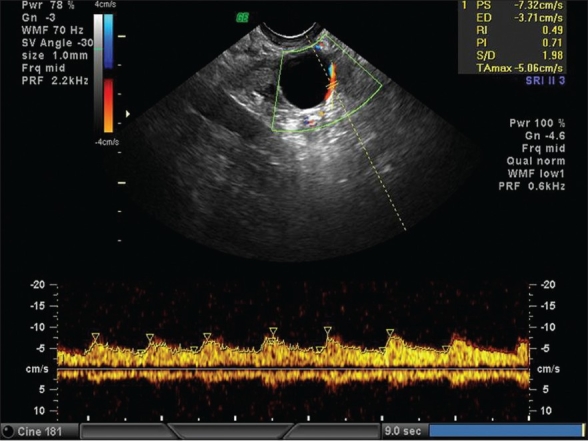
Preovulatory follicle with pulse doppler showing low resistance flow

When a follicle of 16-18 mm was seen on color Doppler, it was considered to be mature enough for hCG injection. But, we have selected the range of resistance index (RI) from 0.4 to 0.56 and peak systolic velocity (PSV) from 7.5 and above as in our patients hCG is given 12 h after the scan [Figures 5–7]. Our study has shown that when the perifollicular RI is >0.53 and the PSV is <9 cm/s, 12 h before hCG injection, the conception rates were only 10.76 and 14.2%, respectively [Table 1, Graphs 1 and 2] as compared with 32.3 and 27%, respectively, and individually when the perifollicular RI was <0.50 and the PSV was >11 cm/s [Table 2, Graphs 3 and 4].

**Figure:**
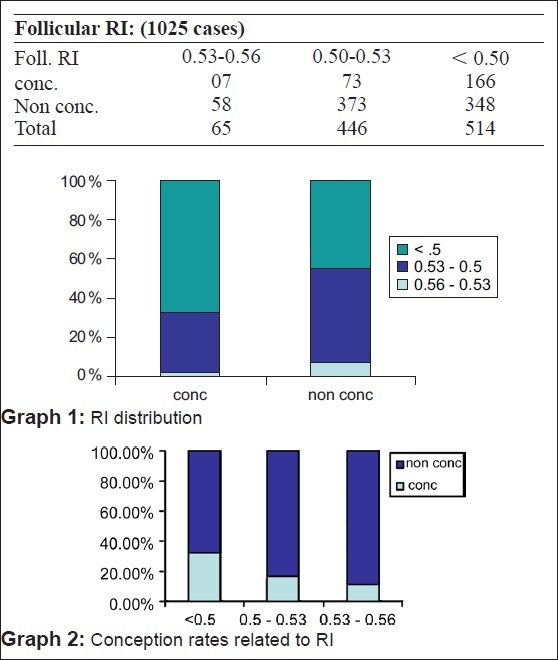


**Figure:**
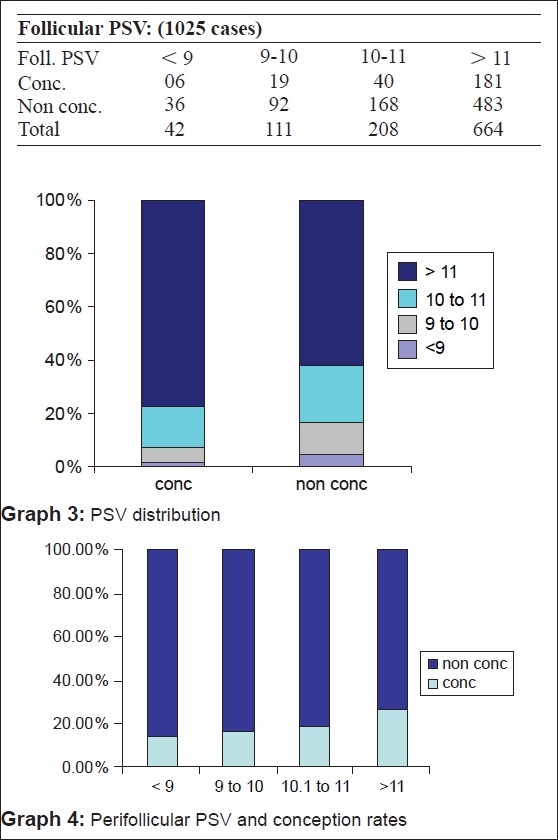


**Figure:**
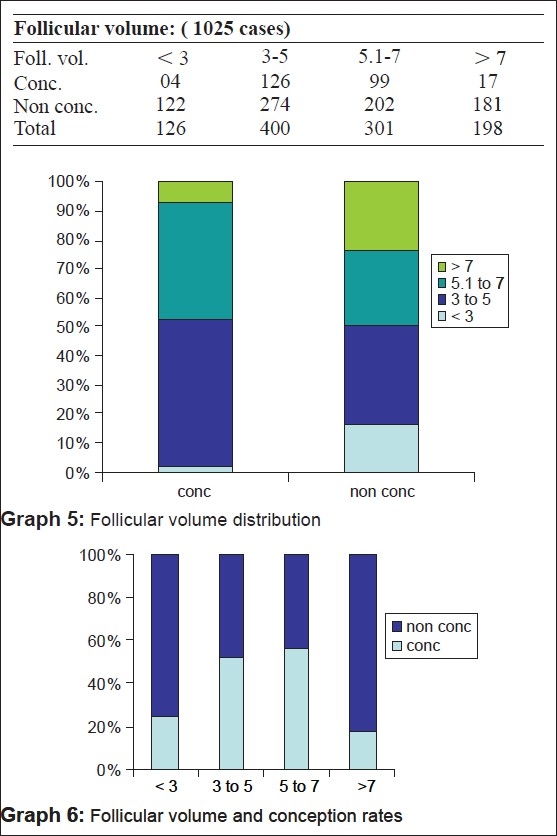


**Figure:**
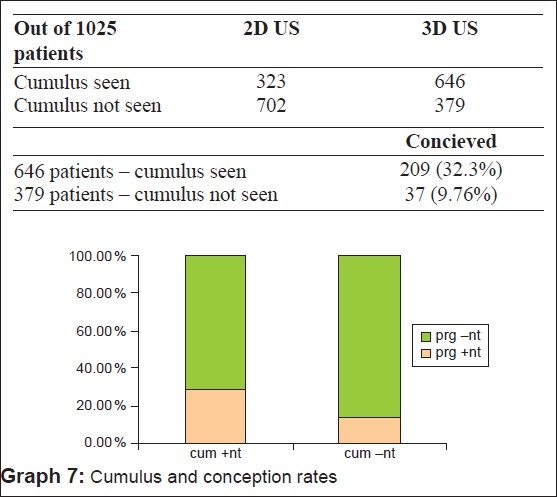


On 3D, the follicular volume of 3-7 cc has been found to be optimum in our study [Table 3 Graphs 5 and 6].

We in this study have been able to locate the cumulus in 94.6% of the cases in conception cycles and in 53% of non-conception cycles by surface rendering [Table 4 and 5, Graph 7].

**Figure:**
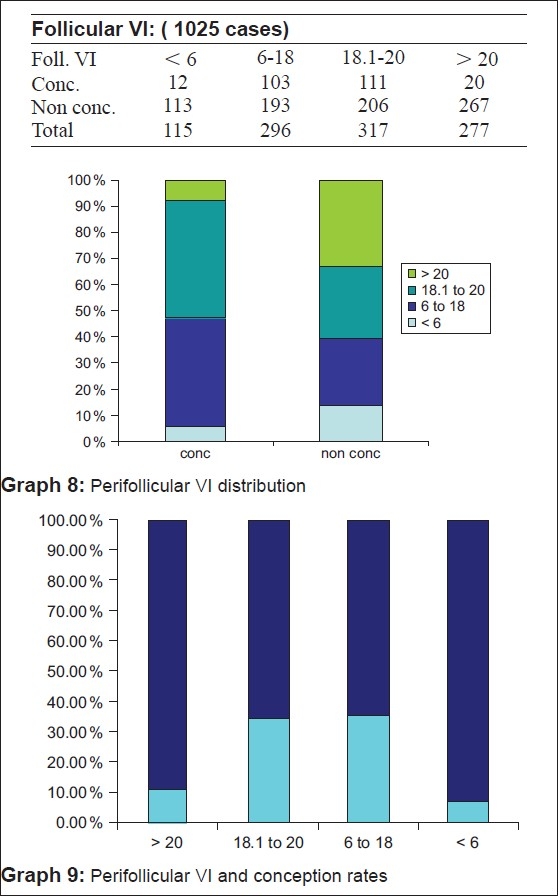


**Figure:**
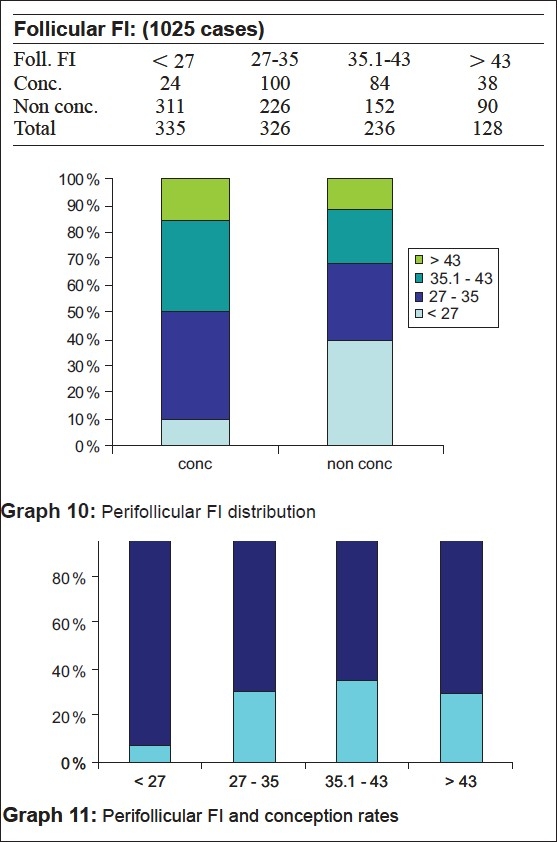


In our study, we have found a perifollicular VI of between 6 and 20 and perifollicular FI > 27 as the most optimum. 34.8% of the patients conceived when the VI was between six and 18 and 35% when it was between 18 and 20. However, the pregnancy rates were <10.4% when VI was <6 and only 7.2% when VI was >20 [Table 6, Graphs 8 and 9]. It was only 7.16% of patients with FI < 27 who conceived whereas it was 30.6% with FI between 27 and 35, 35.5% when FI was between 35 and 43 and 29.6% when FI was >43 [Table 7, Graphs 10 and 11].

## DISCUSSION

The follicular volume achieved in this study varied from 3 to 7.0 cc, which agrees with the study by Witmack *et al*.[3] that states that in *in vitro* fertilization (IVF)–embryo transfer cycles, follicles with a mean follicular diameter of 12-24 mm are associated with optimal rates of oocyte recovery, fertilization and cleavage. This corresponds to the follicular volumes of between 3 and 7 ml. The accuracy of 3D US measurement of follicular volume compared with the standard 2D techniques by comparing the volume of individual follicles is estimated by both methods with the corresponding follicular aspirates: Using the formula of ellipse, the limits of agreement between aspirates and calculated volume were + 3.47 to −2.42 as compared with + 0.96 to −0.43 when calculated by 3D US using[4] the Vocal software. The conception rate for the cumulus seen group was 32.3% as compared with 9.76% only in the cumulus not seen group. Feichtinger *et al*., in their study, have shown the presence of cumulus in follicles >15 mm by 3D US.[5] Follicles without visualization of cumulus in all three planes are not likely to contain mature oocytes. Poehl *et al*. also showed in their study that appearance of the intrafollicular cumulus structures by 3D US was correlated with the recovery rate of mature oocytes.[6] They also found a significant correlation between the number of detected cumuli and the number of retrieved oocytes (*P* < 0.0001), mature oocytes (*P* < 0.0001) and number of fertilized oocytes (*P* < 0.0001). Therefore, visualization of the cumulus by 3D US is a positive indicator of mature oocytes in both IUI and IVF procedures and has been found to be an indicator of successful fertilization in IVF cycles. The follicular fluid concentrations of leptine, a follicular angiogenesis-related factor are inversely related to the stromal blood FI.[7] It has also been suggested that the follicles containing oocytes capable of producing pregnancy have a perifollicular vascular network that is more[8] uniform and distinctive.

Even when the follicle appeared mature according to the 2D US and color and pulse Doppler parameters, the pregnancy rates were significantly better only when the follicular volume was between 3 and 7 cc, cumulus was present and the perifollicular VI was between six and 20 and FI > 27. A study by Kupesic and Kurjak shows that when the ratio of follicular volume to blood FI (FV/FI) is between 0.4 and 0.6, the pregnancy rates are 39%, if > 0.6 it is 52% and when < 0.4 it is only 21%.[9]

## CONCLUSIONS

3D US is much more accurate for volume assessment of the follicle, which is a much more reliable parameter than follicular diameter. The presence of cumulus, which can be confirmed by 3D US, increases the surety of the presence of a mature ovum in the follicle. The 3D PD gives an idea about the global vascularity of the follicle and the endometrium. Recently, 3D volume assessment and 3D PD indices VI, FI and VFI have also been tried in infertility management for assessment of polycystic ovary patients by Pascual *et al*. and have found a strong correlation between 2D and 3D volume as well as 3D PDA indices.[10] Although still larger studies are needed to establish more precise values for follicular VI and FI, the results are fairly promising. Although it is possible to assess the follicular flow as expressed by the PSV and perifollicular color map,[11] it is the 3D PD that proves the most precise information about the vascularization and[12] follicular blood flow.
